# Severity Classification of Conjunctival Hyperaemia by Deep Neural Network Ensembles

**DOI:** 10.1155/2019/7820971

**Published:** 2019-06-02

**Authors:** Hiroki Masumoto, Hitoshi Tabuchi, Tsuyoshi Yoneda, Shunsuke Nakakura, Hideharu Ohsugi, Tamaki Sumi, Atsuki Fukushima

**Affiliations:** ^1^Department of Ophthalmology, Tsukazaki Hospital, Himeji, Japan; ^2^Department of Sensory Science, Kawasaki University of Medical Welfare, Kurashiki, Japan; ^3^Department of Ophthalmology and Visual Science, Kochi Medical School, Nankoku, Japan

## Abstract

Conjunctival hyperaemia is a common clinical ophthalmological finding and can be a symptom of various ocular disorders. Although several severity classification criteria have been proposed, none include objective severity criteria. Neural networks and deep learning have been utilised in ophthalmology, but not for the purpose of classifying the severity of conjunctival hyperaemia objectively. To develop a conjunctival hyperaemia grading software, we used 3700 images as the training data and 923 images as the validation test data. We trained the nine neural network models and validated the performance of these networks. We finally chose the best combination of these networks. The DenseNet201 model was the best individual model. The combination of the DenseNet201, DenseNet121, VGG19, and ResNet50 were the best model. The correlation between the multimodel responses, and the vessel-area occupied was 0.737 (*p* < 0.01). This system could be as accurate and comprehensive as specialists but would be significantly faster and consistent with objective values.

## 1. Introduction

Conjunctival hyperaemia is one of the most common findings in ophthalmologic practice. It is routinely described as a symptom of many ocular diseases such as conjunctivitis, uveitis, elevated intraocular pressure due to glaucoma, and ophthalmic side effects. For example, conjunctival hyperaemia is a minor side effect of glaucoma eye drops, but it becomes relatively important when medication adherence is considered. Most complaints of eye drop-related conjunctival hyperaemia are regarding aesthetics, but patients' dislike of their eyes' appearance can significantly impact their need to continue their medication [1, 2]. Several clinical studies have been conducted to assess conjunctival hyperaemia reactions after glaucoma eye drop instillation [3, 4], but a critical variable in these studies is the determination of the severity of conjunctival hyperaemia.

At present, conjunctival hyperaemia is classified by severity according to the McMonnies and Chapman-Davies scale [5], Institute for Eye Research scale [6], Efron scale [7], a validated bulbar redness scale [8], and the Japan Ocular Allergy Society (JOAS) conjunctival hyperaemia severity grading scale [9]. However, all of these grading systems are purely subjective [10]. In the aforementioned clinical studies, the JOAS system was used; in it, clinicians use standardised photographs to grade the degree of dilation of the conjunctival blood vessels causing hyperaemia on a 4-point scale that includes no hyperaemia. This severity grading is used in clinical studies of the aforementioned glaucoma eye drops [3, 4].

Yoneda et al. invented an analytical application dedicated to conjunctival imaging to establish an objective grading system [11, 12]. In their application, the area occupied by the blood vessels is obtained from images captured by a dedicated conjunctival imaging system. However, Yoneda admits that it is necessary to simplify the application before it can be used in clinical practice [11].

Recently, a supervised machine learning system known as neural network [13] and its algorithms are gaining attention. In particular, in medical research, the deep neural network, which uses many convolution layers [14], has been applied. In ophthalmology, its use has been validated in reports on diabetic retinopathy, glaucoma, age-related macular degeneration, and retinal detachment [15–19]. The imaging devices used to train the machines are also diverse, including a fundus camera, an optical coherence tomographic system, and a wide-angle fundus camera. The advantage of diagnostic and judgement systems using deep learning is the range of their adaptability. For example, using convolutional layers, features can be grasped without the effects of slight noise [20–22]. In addition, although a large amount of computation is required for the learning process, actual grading is performed by a simplified four-rule computation. Thus, a large computing capacity is ultimately unnecessary, and even a small device can be used for verification [23].

Although a clinically useful system that automatically performs hyperaemia grading by deep learning is theoretically possible, to our knowledge, it has not been attempted yet.

Here, we attempted to develop a system that performs as well as ophthalmology specialists using standard slit photographs to teach a deep neural network the conjunctival hyperaemia severity grading of the JOAS.

## 2. Materials and Methods

**Figure 1:**
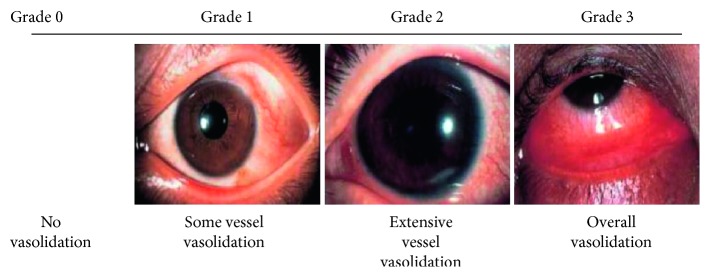
Standard photographs of the severity of conjunctival hyperaemia by Japan Ocular Allergy Society grade. The grading system is defined by the number of dilated vessels in the bulbar conjunctiva. The palpebral conjunctiva is not evaluated.

The Japan Ocular Allergy Society's conjunctival hyperaemia severity grading system (hereafter “JOAS grading”)^9^ is a system to classify the degree of dilation of conjunctival blood vessels in spherical conjunctiva into four levels: none, mild, moderate, and advanced, using a set of standard photographs (Figure 1). This study was performed in accordance with the Declaration of Helsinki. Study protocol and conduct were approved by the Institutional Review Board of Kochi University and Saneikai Tsukazaki Hospital.

### 2.1. Images to Be Analysed

Of all slit lamp photographs taken for clinical purposes at Ophthalmology Department of Tsukazaki Hospital between 01/15/2005 and 07/14/2018, a total of 5,008 photographs were extracted. To make them consistent with the standard JOAS photographs, magnifications of 5× and 8× were used. Slit lamp microscopes by Zeiss Corporation and Hague Straight Corporation were used; the photography conditions such as the amount of light and direction of gaze were not specifically defined. Photographers varied as well. There were no particular inclusion criteria in terms of causative diseases. The patients who have subconjunctival hemorrhage were excluded. Also, images taken after ocular fluorescein staining were included in the analysis.

Excluded from the analysis were all images taken through a cobalt or blue-free philtre. The images not taken under generalised illumination were also excluded. The study was conducted in accordance with the tenets of the Declaration of Helsinki. Study protocol and conduct were approved by the Institutional Review Board of Kochi University and Saneikai Tsukazaki Hospital.

### 2.2. Image Data

**Figure 2:**
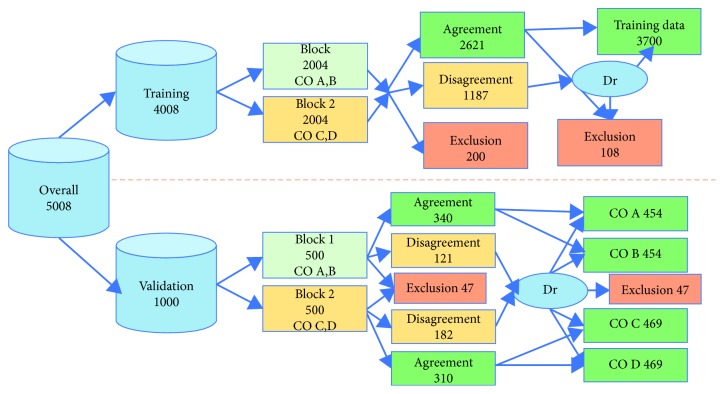
Image data flow. The top branch represents data flow for creating an artificial intelligence model. The bottom branch represents data flow for preliminary grader evaluation and model evaluation. CO was a certified orthoptist (expert grader); Dr was a doctor who is a specialist in both the Japanese Society of Allergology and the Japanese Ophthalmological Society (specialist grader). The data flow processes for training data and evaluation data were different because defining correct responses required different protocols for each process.

The initial 5,008 images were divided into two groups: 4,008 images for preparing the artificial intelligence model (hereafter “for training”) and 1,000 images for preliminary validation by graders and for model validation (hereafter “for validation”). An overview of the data flow for training and subsequent validation is provided in Figure 2; details will be described in the appropriate sections below.

### 2.3. Selection of Graders

In this study, the quadratic-weighted kappa coefficient [24] was used to first examine interrater agreement for the 1,000 validation images. This allowed us to determine the quality of responses and evaluate performance by excluding coincidence rates (chance positive results due to data distribution) [24].

JOAS grading was performed by a physician who was a specialist member of the Japanese Society of Allergology and the Japanese Ophthalmological Society (hereafter the “specialist”), as well as four certified orthoptists (COs). The 1,000 validation images were graded individually and completely independently (i.e., no consultations among graders). Five images were excluded due to mistakes, and those images considered to be ungradable by at least one grader were excluded when calculating the weighted kappa coefficient. As a result, a total of 881 images were included in this analysis.

As shown in Table 1, all 4 COs and the specialist graded with weighted kappa coefficients of above 0.7; therefore, they were considered grading experts (hereafter “the experts”) for the purposes of reference (correct) JOAS grading scores during training.

### 2.4. Training Data

The 4,008 training images were randomly divided into two sets of 2,004 images. Two COs (A and B or C and D) graded one image set each using JOAS grading. Images considered to be ungradable by either of the two during the grading process were excluded from analysis. In addition, some images were lost due to data management errors. A total of 200 images were lost from the full training image set. For the remaining 3,808 images, both graders were in agreement for 2,621 images; this set was then used for the training data. The remaining 1,187 images which were inconsistent in grades were graded again by the specialist, who reinstated a total of 1,079 of the inconsistent images. As a result, a total of 3,700 images were included in the training data.

### 2.5. Validation Data

One thousand images were randomly divided into two sets of 500 images to be used for validation of the system. For each set of 500 images, responses used for the selection of graders were adopted as experts' responses to be used for performance evaluation of the artificial intelligence model. The images with consistent responses were adopted as experts' responses (there were no consistent responses for ungradable images), and some of the images with inconsistent responses were excluded from analysis by the specialist. For those images with inconsistent answers, if they were not excluded from analysis, the experts' responses were adopted. For validation data, 454 images were included for CO A and B and 469 images for CO C and D. There were 923 images in total.

### 2.6. Building of the Artificial Intelligence Model

The grade classification of the training data was as follows: Grade 0, 688 images; Grade 1, 1734; Grade 2, 1176, and Grade 3, 102. Image processing was performed on the training data to amplify images as follows: Grade 0, 4 times; Grade 1 and 2, doubled; Grade 3, 18 times. Doing this allowed us to have smaller differences on the number of training images after data expansion between grades. In the image amplification processing, inversion was always carried out. Other processing performed included no corrections, contrast adjustment (increase or decrease), *γ* correction (*γ* = 0.75 or 1.5), histogram equalisation, Gaussian noise addition, and salt and pepper noise addition. Of the nine types of processing employed, the types of processing to be performed on each grade were randomly chosen: 2 types for Grade 0, 1 type for Grade 1, 1 type for Grade 2, and 9 types for Grade 3. With the amplified images, we trained nine types of network structures (VGG16, VGG19, ResNet50, InceptionV3, InceptionResNetV2, Xception, DenseNet121, DenseNet169, and DenseNet201) [25–30] and built nine models. Using each model, we tested the validation data and evaluated the model.

### 2.7. Deep Learning Model and Training

The VGG16 network structure can be divided into five binding blocks composed of a convolution layer and max pooling layer, as well as the fully connected layer [25].

First, all input images were converted to 256 × 192 pixels in advance, read in 8-bit RGB colour and 256 pixels ∗ 192 pixels ∗ 3 channels tensors. The input was normalised to the range of 0-1 by dividing it by 255.

The convolutional layer recognises features of the target through convolutional filters [20–22]. The max pooling layer was placed at the end of each block; it reduces the position sensitivity of a feature output from a convolutional layer so that a more general recognition can be performed [31].

Finally, after flattening the three-dimensional matrix, we arranged two layers of the fully connected layer and classified them into four classes by a softmax function. The purpose of the fully connected layer is to remove spatial information from extracted features and to statistically distinguish the target from other feature vectors [32]. Dropout processing was applied to the first fully connected layer to mask out with 50% probability. The purpose of dropout processing is to improve the generalisation performance and prevent overlearning during the training [33].

As an output, the probability distribution for the outcome of the sum becoming 1 is displayed, making the item with the largest value as the output grade.

We used a method called fine-tuning, which uses already-learned parameters with different data. Its objective is to increase the training speed and easily obtain high performance even with a small amount of data [34]. The parameters obtained from learning Imagenet were used as initial values of the parameters for layers other than the fully connected layer, and the training was conducted to obtain appropriate parameters from the initial values.

The initial weight update was performed according to an optimisation algorithm called Momentum SGD (learning coefficient = 0.001, inertia term = 0.9), which is one of the stochastic gradient descent methods [35, 36]. Categorical cross-entropy was used for a loss function.

Also, each grade was given a different weight for the loss function.  Table 2 lists the weight for each grade.

Fine-tuning was performed on other network structures as well. For layers except the fully connected layer, we used parameters by learning Imagenet as initial values, and learning and validation of classification were done using two fully connected layers and a dropout layer. The optimiser and loss function are the same as in the case of VGG16.

The construction and validation of the model were carried out using Keras (https://keras.io/en/) which runs Python's TensorFlow backend (https://www.tensorflow.org/). The training and validation of the model were done using the GeForce GTX 1080 Ti GPU by NVIDIA.

### 2.8. Performance Evaluation in a Single Model

Performance evaluation in each model was performed using the weighted kappa coefficient.

Of all the validation data, we set the weighted kappa coefficients of CO A and CO B as *κ*_ab_ for data using the responses by CO A and B (*κ*_na_ and *κ*_nb_, respectively). Likewise, CO C and CO D were set as *κ*_cd_, *κ*_nc_, and *κ*_nd_. Here, we set and calculated the evaluation index called kappa distance score (KDS) to find how close the responses of the model were to those of humans.

(1) $$\begin{array}{c} \text{KDS}=\left(\kappa_{\text{na}}+\kappa_{\text{nb}}-2\kappa_{\text{ab}}\right)+\left(\kappa_{\text{nc}}+\kappa_{\text{nd}}-2\kappa_{\text{cd}}\right). \end{array}$$

### 2.9. Comparison of Kappa Coefficients between Models

For the total of 923 validation images, the weighted kappa coefficients of each model were compared to examine whether any of the models provided a different response. In comparison with other models, we excluded those with an average of weighted kappa coefficient of 0.7 or less and those with the lowest average of weighted kappa coefficients.

This was done until there were no more models with an average of weighted kappa coefficients of 0.7 or less in comparison with other models, and only the remaining models were used as the models for performance evaluation in a multimodel.

### 2.10. Performance Evaluation in a Multimodel State

When using a multimodel, there are two ways to set which of the neural network's responses are to be used:

1. If the responses of more than half of the models of *N* (number) models match with the expert's responses, the responses are considered the same; otherwise, the grade with the highest number of models' responses are considered as the responses of the multimodel.
2. If the responses of at least one model of *N* (number) models match with the expert's responses, the responses are considered the same; otherwise, the grade with the highest number of models' responses are considered as the responses of the multimodel.

Based on the responses of (1), the score obtained by the method described in the performance evaluation of a single model was set as KDS_(*n*,half)_ and responses obtained in (2) were set as KDS_(*n*,least)_.

Seven out of nine models were used for validation. In the order of the highest KDS when performing the single-model performance evaluation for each of the seven models, KDS_(*n*,half)_ and KDS_(*n*,least)_ at *N* = 2 to 7 were calculated using *N* models.

Also, KDS_(*n*,half)_ and KDS_(*n*,least)_ values were normalised so that the average would equal 0 and the standard deviation would equal 1, and the total value was set as KDS_(*n*,multi)_ as shown below:

(2) $$\begin{array}{c} {\text{KDS}}_{\left(n,\text{multi}\right)}=\frac{{\text{KDS}}_{\left(n,\text{half}\right)}-\text{average}\left({\text{KDS}}_{\left(\text{half}\right)}\right)}{\text{SE}\left({\text{KDS}}_{\left(\text{half}\right)}\right)}+\frac{{\text{KDS}}_{\left(n,\text{least}\right)}-\text{average}\left({\text{KDS}}_{\left(\text{least}\right)}\right)}{\text{SE}\left({\text{KDS}}_{\left(\text{least}\right)}\right)},\\ \text{average}\left({\text{KDS}}_{\left(\text{half}\right)}\right)=\;\frac{1}{6}\overset{7}{\underset{k=2}{\sum }}{\text{KDS}}_{\left(k,\text{half}\right)},\\ \text{SE}\left({\text{KDS}}_{\left(\text{half}\right)}\right)=\sqrt{\frac{1}{6}\overset{7}{\underset{k=2}{\sum }}\left({\text{KDS}}_{\left(k,\text{half}\right)}\right)-\text{average}{\left({\text{KDS}}_{\left(\text{half}\right)}\right)}^{2}},\\ \text{average}\left({\text{KDS}}_{\left(\text{least}\right)}\right)=\frac{1}{6}\overset{7}{\underset{k=2}{\sum }}{\text{KDS}}_{\left(k,\text{least}\right)},\\ \text{SE}\left({\text{KDS}}_{\left(\text{least}\right)}\right)=\sqrt{\frac{1}{6}\overset{7}{\underset{k=2}{\sum }}\left({\text{KDS}}_{\left(k,\text{least}\right)}\right)-\text{average}{\left({\text{KDS}}_{\left(\text{least}\right)}\right)}^{2}}. \end{array}$$

### 2.11. Quantitative Evaluation

**Figure 3:**
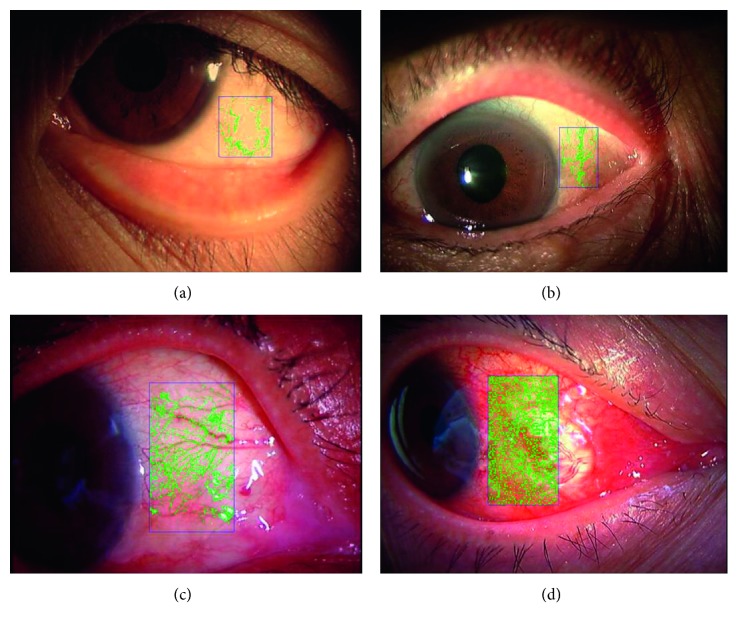
Software to measure the area occupied by blood vessels. It calculates the area occupied by blood vessels in terms of a per-pixel threshold value (the green overlay in the images demonstrates above-threshold (positive) values) and outputs that as a percentage of imaged area (the box). (a) 5.2%, (b) 9.5%, (c) 13.0%, (d) 33.4%.

We compared the responses obtained by the software to the image area occupied by blood vessels in the bulbar conjunctiva as developed by Yoneda et al. [11, 12] with the responses by the multimodel. The evaluation screen is shown in Figure 3.

To obtain the multimodel's response, grades provided by the 6-model multimodel were averaged and rounded. Except for Grade 3, images were randomly selected.

We investigated whether there is a correlation between the response of the multimodel and the area occupied, as well as whether there is a significant difference in the area occupied by blood vessels per each response grade of the multimodel.

### 2.12. Statistical Analysis

We used a Python module called scikit-learn to calculate the weighted kappa coefficients. Although the confidence interval can be obtained from approximate standard error for the weighted kappa coefficients [24], there is no established calculation method for confidence intervals such as KDS using multiple weighted kappa coefficients. Therefore, there is no established way to test whether the score exceeds zero.

It is also difficult to examine whether there is a significant difference in score per model. For the above reasons, calculation of confidence intervals and statistical test were not performed for each statistic.

Spearman's rank-correlation test [37] was performed to examine the correlation between Yoneda's software and the multimodel. The Kruskal–Wallis [38] and Steel–Dwass [39] tests were conducted to investigate whether there was a significant difference in the area occupied by blood vessels of each response grade of the multimodel.

## 3. Results and Discussion

For each model, we trained the neural network to grade conjunctival hyperaemia using the JOAS system with 3,700 images. We then used 923 other images as validation test images to evaluate how well the model could grade. The average age and female ratio of training images and validation test images were 50.6 (±21.2) year and 57.0%. We calculated weighted kappa coefficients (*κ*) as interrater reliability measures for the experts (Table 1) and then for each of the models (Table 3). We then used these values to calculate a kappa distance score (KDS) for each model, as well as for a multimodel system in which two or more of the seven best models were combined to give a single final output (i.e., each model in the multimodel got a “vote,” and the votes were tallied for the “winning” grade ouptut). The KDS represents how close the model's responses matched the expert clinicians' responses, such that higher values are a closer match and values above zero are considered clinically acceptable.

### 3.1. Single-Model Evaluation

Table 3 shows *κ* scores and KDSs for each model. We found that the DenseNet201 model was the best individual model (i.e., highest KDS); however, no individual model reached a KDS above zero.

Evaluation of intermodel kappas for multimodel inclusion.

The weighted kappa coefficients between models were also calculated. The average kappa coefficients of the InceptionResNetV2 model and Xception model were below the acceptable threshold of 0.7 and were the two lowest (Supplementary Tables 1 and 2). Thus, they were excluded and the following seven models (Supplementary Table 3) were evaluated for performance in a multimodel system: DenseNet201, DenseNet121, VGG19, DenseNet169, VGG16, ResNet50, and InceptionV3.

### 3.2. Multimodel Evaluation

**Figure 4:**
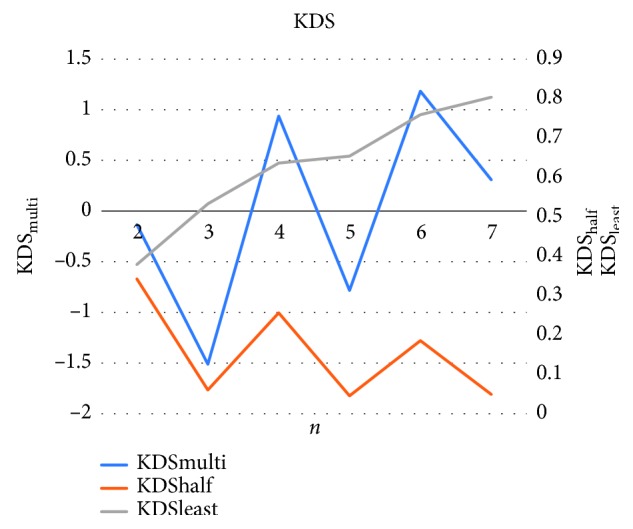
Multimodel KDS values for *n* = 2 to 7 models in the system. The left *y*-axis indicates KDS_(*n*,multi)_; the right *y*-axis indicates KDS_(*n*,half)_ and KDS_(*n*,least)_. The “n” on the horizontal axis indicates the number of models being used in that combination.

Figure 4 shows the three KDS values assessed for each multimodel system (*n* = 2 to 7 included models): KDS_(*n*,half)_, KDS_(*n*,least)_, and KDS_(*n*,multi)_. Of these, KDS_(*n*,half)_ represents diagnostic accuracy (the ability to score the correct grade), KDS_(*n*,least)_ represents diagnostic completeness (the ability to provide at least one correct response when faced with several correct options), and KDS_(*n*,multi)_ represents the net KDS score (how close the system was to the experts). KDS_(*n*,half)_ was above zero for all systems, indicating that when combined, the individual models can achieve clinically acceptable diagnostic accuracy. KDS_(*n*,multi)_ was highest for the 6-model system, suggesting that combining the DenseNet201, DenseNet121, VGG19, DenseNet169, VGG16, and ResNet50 models produces the best overall outcome (Supplemental Tables 4).

**Figure 5:**
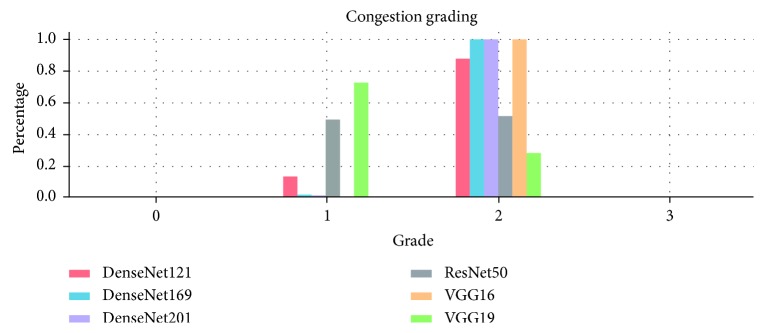
An example of the six-model multimodel grading. Using JOAS grades, four models of the six provided the response of Grade 2 (DenseNet121, DenseNet169, DenseNet201, and VGG16), whereas two models provided the response of Grade 1 (ResNet50 and VGG19). Therefore, the output was “Grade 2”.

An example of a response provided by an actual model is shown in Figure 5. It took 411.0 seconds to generate the graph of the responses for the 923 test images, which translates to a scoring speed of 0.445 seconds per image.

### 3.3. Correlation with Quantitative Data

**Figure 6:**
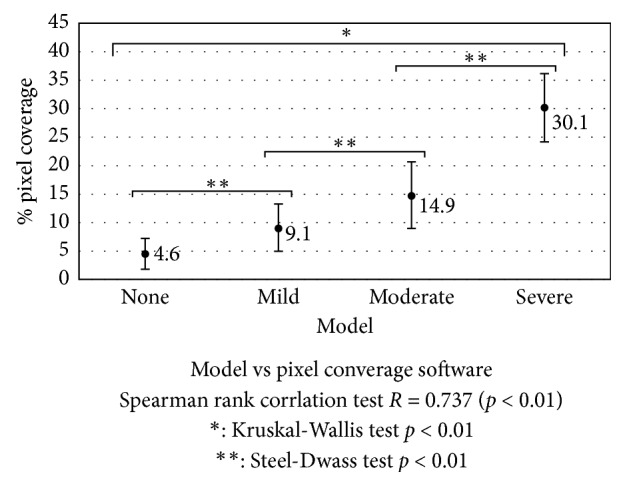
Comparison of responses of multimodel and the area occupied by blood vessels. There is a correlation between the responses of the multimodel system and the area occupied by blood vessels. Also, there is a significant difference in the area occupied by blood vessels between all grades.

Figure 6 shows the multimodel's responses, and the area occupied by blood vessels measured with Yoneda et al.'s software (Supplementary Table 5). Their software was able to measure the area occupied by blood vessels for 71.8% of all images. The correlation between the multimodel responses and the vessel-area occupied was 0.737 (*p* < 0.01). In addition, significant differences were found between each pair of grades when comparing the measured areas occupied by grade, as well as between grade pairs when comparing the multimodel responses (*p* < 0.01). This suggests that both the software and the multimodel system can distinguish clearly between grades and that the area of an image occupied by blood vessels can be a quantitative marker for each grade.

## 4. Discussion

In this study, we developed a deep learning system that grades the severity of hyperaemia with a high degree of consistency with expert graders and can do so with objective criteria (image area occupied by blood vessels). Hyperaemia grading can have different responses. When using the neural network, or supervised learning, it is necessary to teach input (image) and output (grading) at the same time [13]. Therefore, for training purposes, it is necessary to know the correct response for each image, so we used the experts' responses as the correct responses for images when they agreed and the specialist's response when the experts disagreed. For validation, however, it is not required to teach the correct response, so the experts' responses were used to assess responses. Thus, the data flow differed between the validation data and the training data systems. KDS_(*n*,half)_ represents the accuracy of diagnosis, and KDS_(*n*,least)_ represents the completeness of diagnosis. In other words, a diagnosis would be inaccurate if a system provided random responses across grades; that would be clinically unacceptable. At the same time, when various diagnoses (correct options) are possible, the system could provide responses that do not include any correct response grades, which would be problematic in terms of risk management. The grading by experts can halve this problem. Because KDS_(*n*,half)_ was greater than 0 in the 6-model multimodel system, it can be considered to have clinically acceptable accuracy. Because the KDS_(*n*,multi)_ score was the highest, it is also considered the most comprehensive diagnostic model among all the combinations of models examined in this study. KDS_(*n*,half)_ is markedly lower in the *n* = 3, 5, and 7 systems. This is because when *n* is an even number, “more than half” of the models have to provide a correct response to score as correct; on the other hand, when *n* is an odd number, the “majority” of the models have to provide a correct response to score as correct. That is, in odd-numbered combinations, one more model must be in agreement than in their even-numbered (i.e., one less model/combination) counterparts; this reduces the likelihood of success, which is represented by a lower KDS.

JOAS grading is used as a subjective indicator by doctors in both research and clinical practice [9]. On the other hand, the area occupied by blood vessels in an image has been applied by Yoneda et al. [11] as an objective indicator in research studies. The multimodel system created in this study was highly consistent with both the subjective and objective indicators. One might think that it would be best to use the area occupied by blood vessels as an objective indicator in clinical practice, but there is a drawback to doing so. Continuous values (those represented by a range of numbers rather than discreet categories) are rarely used as a basis for decision-making. Far more often, one or more threshold values are set to perform categorical classification. Because the vessel-occupied area is continuous, subjective thresholds must be set, reducing the objectivity of the value. Thus, it is as meaningful (or more so) for a neural network to use the categorical value, grading, instead of measuring the area occupied by blood vessels. Given these conditions, we believe that our multimodel system, particularly the 6-model system, matches the subjective performance of JOAS grading by clinicians and is, therefore, a clinically relevant model.

One of the strengths of this multimodel system is that it was created using images acquired in routine clinical practice. Thus, the system does not require imaging methods specific for grading hyperaemia or special imaging devices; instead, it can use images acquired using a standard slit lamp microscope with adjustable angle and magnification, suggesting that it is highly suitable for routine clinical application. For example, a patient being seen for corneal foreign bodies (e.g., pieces of metal) could have associated hyperaemia automatically graded at the same time. This would allow the ophthalmologist to evaluate improvement of the hyperaemia at the time of follow-up for the corneal inclusions. With our system, it is possible to improve the quality of care easily with software and without adding any special equipment. In fact, the software used to measure the area occupied by blood vessels was able to measure only about 70% of all the data used in this study.

There are several limitations to our prototype system. First, our study results suggest that it would be necessary to clearly illustrate the neural networks' focus area in clinical practice. At present, the neural network should only be used to assist physicians in making a final decision, instead of allowing it to make an independent diagnosis. Therefore, it is important to include a function in the system wherein the relevant part of the reasoning used by the neural network is directly communicated to the physician; in fact, a few recent medical papers have addressed one such form of reasoning, segmentation [40, 41]. In our system, however, clinically produced hyperaemia images are used, and these will be seen by the physician as part of his or her examination. This obviates the need for that communicative function. Second, this study is a retrospective data search within a single facility, and it is necessary to evaluate the robustness of the model by conducting a prospective study on data from one or more other facilities.

## 5. Conclusions

In this study, we developed an artificial intelligence-based grading system that was accurate and highly consistent with grading by clinical experts. We would like to develop this system further by improving the problematic aspects mentioned above, utilising it in an actual clinical setting and adding the necessary functions to allow it to be applied in much more widespread applications.

## Figures and Tables

**Table 1:** Weighted kappa coefficients of one Japan Ocular Allergy Society-certified specialist and four certified orthoptists.

Opponent	CO A	CO B	CO C	CO D
Dr1	0.727	0.717	0.717	0.749

All COs had weighted kappa coefficients above 0.7. CO: certified orthoptist. Dr1: doctor who is a specialist of both the Japanese Society of Allergology and the Japanese Ophthalmological Society.

**Table 2:** Weighting per grade.

	Grade 0	Grade 1	Grade 2	Grade 3
Weight	1.5	1	1	5

Weighting parameters were set based on the balance between the amount of data and the importance of diagnosis.

**Table 3:** Weighted kappa coefficients and kappa distance scores (KDS) for each model and expert graders.

Model	Κna	Κnb	Κab	Κnc	Κnd	Κcd	KDS
DenseNet201	0.693	0.707	0.748	0.712	0.643	0.653	−0.048
DenseNet121	0.693	0.713		0.704	0.642		−0.051
VGG19	0.692	0.708		0.714	0.637		−0.052
DenseNet169	0.656	0.690		0.718	0.663		−0.077
VGG16	0.679	0.692		0.681	0.651		−0.100
ResNet50	0.685	0.713		0.678	0.614		−0.114
InceptionV3	0.655	0.677		0.655	0.550		−0.266
Xception	0.580	0.669		0.619	0.538		−0.397
InceptionResNetV2	0.576	0.625		0.571	0.515		−0.517

Table is arranged in the order of the higher KDS in the evaluation of models. Κna: the weighted kappa coefficients of neural network and CO A. Κnb: the weighted kappa coefficients of neural network and CO B. Κab: the kappa coefficients of CO A and CO B. Κnc: the kappa coefficients of the neural network and CO D. Κnd: the kappa coefficients of the neural network and CO C. Κcd: the kappa coefficients of CO C and CO D. KDS: kappa scores.

## Data Availability

The clinical data used for the training and validation sets were collected at Tsukazaki Hospital. They are not publicly available as restrictions apply to their use. The data are available with corresponding author on reasonable request.

## Supplementary Materials

Supplementary Materials Supplementary Tables 1–3: the average kappa coefficients of each model and the other models. Supplementary Table 4: multimodel combinations and kappa distance scores (KDS) for *n* = 2 to 7 models/combination. Supplementary Table 5: responses of the multimodel system by grade and the area occupied by blood vessels.

## References

[1] Inoue K. (2014). Managing adverse effects of glaucoma medications. *Clinical Ophthalmology*.

[2] Friedman D. S., Hahn S. R., Gelb L. (2008). Doctor-patient communication, health-related beliefs, and adherence in glaucoma. *Ophthalmology*.

[3] Yanagi M., Kiuchi Y., Yuasa Y. (2016). Association between glaucoma eye drops and hyperemia. *Japanese Journal of Ophthalmology*.

[4] Terao E., Nakakura S., Fujisawa Y. (2017). Time course of conjunctival hyperemia induced by a rho-kinase inhibitor anti-glaucoma eye drop: ripasudil 0.4%. *Current Eye Research*.

[5] McMonnies C. W., Chapman-Davies A. (1987). Assessment of conjunctival hyperemia in contact lens wearers: part I. *Optometry and Vision Science*.

[6] Institute for Eye Research (2007). *IER Grading Scales*.

[7] Efron N. (1997). Clinical application of grading scales for contact lens complications. *Optician*.

[8] Schulze M. M., Jones D. A., Simpson T. L. (2007). The development of validated bulbar redness grading scales. *Optometry and Vision Science*.

[9] Takamura E., Uchio E., Ebihara N. (2017). Japanese guidelines for allergic conjunctival diseases 2017. *Allergology International*.

[10] Schulze M. M., Hutchings N., Simpson T. L. (2008). The use of fractal analysis and photometry to estimate the accuracy of bulbar redness grading scales. *Investigative Opthalmology & Visual Science*.

[11] Yoneda T., Sumi T., Takahashi A., Hoshikawa Y., Kobayashi M., Fukushima A. (2012). Automated hyperemia analysis software: reliability and reproducibility in healthy subjects. *Japanese Journal of Ophthalmology*.

[12] Sumi T., Yoneda T., Fukuda K. (2013). Development of automated conjunctival hyperemia analysis software. *Cornea*.

[13] Carpenter G. A., Grossberg S., Markuzon N., Reynolds J. H., Rosen D. B. (1992). Fuzzy ARTMAP: a neural network architecture for incremental supervised learning of analog multidimensional maps. *IEEE Transactions on Neural Networks*.

[14] LeCun Y., Bengio Y., Hinton G. (2015). Deep learning. *Nature*.

[15] Gulshan V., Peng L., Coram M. (2016). Development and validation of a deep learning algorithm for detection of diabetic retinopathy in retinal fundus photographs. *Journal of the American Medical Association (JAMA)*.

[16] Ohsugi H., Tabuchi H., Enno H., Ishitobi N. (2017). Accuracy of deep learning, a machine-learning technology, using ultra—wide-field fundus ophthalmoscopy for detecting rhegmatogenous retinal detachment. *Scientific Reports*.

[17] Matsuba S., Tabuchi H., Ohsugi H. (2018). Accuracy of ultra-wide-field fundus ophthalmoscopy-assisted deep learning, a machine-learning technology, for detecting age-related macular degeneration. *International Ophthalmology*.

[18] Hiroki M., Tabuchi H., Nakakura S., Naofumi I., Miki M., Hiroki E. (2018). Deep-learning classifier with an ultrawide-field scanning laser ophthalmoscope detects glaucoma visual field severity. *Journal of Glaucoma*.

[19] Sonobe T., Tabuchi H., Ohsugi H. (2018). Comparison between support vector machine and deep learning, machine-learning technologies for detecting epiretinal membrane using 3D-OCT. *International Ophthalmology*.

[20] Deng J., Dong W., Socher R., Li L.-J., Li K., Fei-Fei L. Imagenet: a large-scale hierarchical image database.

[21] Russakovsky O., Deng J., Su H. (2015). Imagenet large scale visual recognition challenge. *International Journal of Computer Vision*.

[22] Lee C. Y., Xie S., Gallagher P., Zhang Z., Tu Z. Deeply-supervised nets.

[23] Amato G., Carrara F., Falchi F., Gennaro C., Meghini C., Vairo C. (2017). Deep learning for decentralized parking lot occupancy detection. *Expert Systems with Applications*.

[24] Heo M. (2008). Utility of weights for weighted kappa as a measure of interrater agreement on ordinal scale. *Journal of Modern Applied Statistical Methods*.

[25] Simonyan K., Andrew Z. (2014). Very deep convolutional networks for large-scale image recognition. https://arxiv.org/pdf/1409.1556.pdf.

[26] He K., Zhang X., Ren S., Sun J. Deep residual learning for image recognition.

[27] Huang G., Liu Z., Van Der Maaten L., Weinberger K. Q. Densely connected convolutional networks.

[28] Szegedy C., Vanhoucke V., Ioffe S., Shlens J., Wojna Z. Rethinking the inception architecture for computer vision.

[29] Szegedy C., Ioffe S., Vanhoucke V., Alemi A. A. Inception-v4, inception-resnet and the impact of residual connections on learning.

[30] Chollet F. (2017). Deep learning with depthwise separable convolutions. https://arxiv.org/pdf/1610.02357.pdf.

[31] Scherer D., Müller A., Behnke S. Evaluation of pooling operations in convolutional architectures for object recognition.

[32] Krizhevsky A., Sutskever I., Hinton G. E. (2012). Imagenet classification with deep convolutional neural networks. *Advances in Neural Information Processing Systems*.

[33] Srivastava N., Hinton G., Krizhevsky A., Sutskever I., Salakhutdinov R. (2014). Dropout: a simple way to prevent neural networks from overfitting. *Journal of Machine Learning Research*.

[34] Agrawal P., Girshick R., Malik J. Analyzing the performance of multilayer neural networks for object recognition.

[35] Qian N. (1999). On the momentum term in gradient descent learning algorithms. *Neural Networks*.

[36] Nesterov Y. (1983). A method for unconstrained convex minimization problem with the rate of convergence O (1/k^2^). *Doklady AN USSR*.

[37] Zar J. H. (1972). Significance testing of the Spearman rank correlation coefficient. *Journal of the American Statistical Association*.

[38] Theodorsson-Norheim E. (1986). Kruskal-Wallis test: BASIC computer program to perform nonparametric one-way analysis of variance and multiple comparisons on ranks of several independent samples. *Computer Methods and Programs in Biomedicine*.

[39] Ishida Y., Nakamura F., Kanzato H. (2005). Clinical effects of *Lactobacillus acidophilus* strain L-92 on perennial allergic rhinitis: a double-blind, placebo-controlled study. *Journal of Dairy Science*.

[40] González Sánchez J. C. (2018). *Segmentation of Bones in Medical Dual-Energy CT Volumes Using the 3D U-Net Convolutional Neural*.

[41] Li X., Chen H., Qi X., Dou Q., Fu C. W., Heng P. A. (2017). H-DenseUNet: hybrid densely connected UNet for liver and liver tumor segmentation from CT volumes. https://arxiv.org/pdf/1709.07330.pdf.

