# Supplementary material for: Severity Classification of Conjunctival Hyperaemia by Deep Neural Network Ensembles
Source: J Ophthalmol. 2019 Jun 2;2019:7820971. doi: 10.1155/2019/7820971 (PMC6589312; doi:10.1155/2019/7820971)
Supplement: Supplementary Materials — Supplementary Tables 1–3: the average kappa coefficients of each model and the other models. Supplementary Table 4: multimodel combinations and kappa distance scores (KDS) for n = 2 to 7 models/combination. Supplementary Table 5: responses of the multimodel system by grade and the area occupied by blood vessels. [file 7820971.f1.docx]

**Supplementary Tables**

**Supplementary Table 1**. The average kappa coefficients of the InceptionResNetV2 and other models was below the acceptable threshold of 0.7 and was the lowest.

| Opponent | DenseNet121 | DenseNet169 | DenseNet201 | InceptionResNetV2 | InceptionV3 | ResNet50 | VGG16 | VGG19 | Xception | average |
| --- | --- | --- | --- | --- | --- | --- | --- | --- | --- | --- |
| DenseNet121 |  | 0.780858159 | 0.791819015 | 0.655955395 | 0.708545801 | 0.742686271 | 0.764111097 | 0.753960128 | 0.701109835 | 0.737380713 |
| DenseNet169 | 0.780858159 |  | 0.798056885 | 0.648175677 | 0.703626402 | 0.758786197 | 0.780587595 | 0.771494253 | 0.695546796 | 0.742141496 |
| DenseNet201 | 0.791819015 | 0.798056885 |  | 0.675635309 | 0.708621525 | 0.744189332 | 0.796563813 | 0.805089111 | 0.673512045 | 0.74918588 |
| InceptionResNetV2 | 0.655955395 | 0.648175677 | 0.675635309 |  | 0.650131116 | 0.662133323 | 0.66250371 | 0.667611581 | 0.682746192 | 0.663111538 |
| InceptionV3 | 0.708545801 | 0.703626402 | 0.708621525 | 0.650131116 |  | 0.710686588 | 0.709433337 | 0.710539441 | 0.684172216 | 0.698219553 |
| ResNet50 | 0.742686271 | 0.758786197 | 0.744189332 | 0.662133323 | 0.710686588 |  | 0.74705286 | 0.743972582 | 0.70292887 | 0.726554503 |
| VGG16 | 0.764111097 | 0.780587595 | 0.796563813 | 0.66250371 | 0.709433337 | 0.74705286 |  | 0.826759506 | 0.684326475 | 0.746417299 |
| VGG19 | 0.753960128 | 0.771494253 | 0.805089111 | 0.667611581 | 0.710539441 | 0.743972582 | 0.826759506 |  | 0.665797527 | 0.743153016 |
| Xception | 0.701109835 | 0.695546796 | 0.673512045 | 0.682746192 | 0.684172216 | 0.70292887 | 0.684326475 | 0.665797527 |  | 0.686267495 |

**Supplementary Table 2**. The average kappa coefficients of the Xception and other models was below the acceptable threshold of 0.7 and was the lowest.

| Opponent | DenseNet121 | DenseNet169 | DenseNet201 | InceptionV3 | ResNet50 | VGG16 | VGG19 | Xception | average |
| --- | --- | --- | --- | --- | --- | --- | --- | --- | --- |
| DenseNet121 |  | 0.780858159 | 0.791819015 | 0.708545801 | 0.742686271 | 0.764111097 | 0.753960128 | 0.701109835 | 0.749012901 |
| DenseNet169 | 0.780858159 |  | 0.798056885 | 0.703626402 | 0.758786197 | 0.780587595 | 0.771494253 | 0.695546796 | 0.755565184 |
| DenseNet201 | 0.791819015 | 0.798056885 |  | 0.708621525 | 0.744189332 | 0.796563813 | 0.805089111 | 0.673512045 | 0.759693104 |
| InceptionV3 | 0.708545801 | 0.703626402 | 0.708621525 |  | 0.710686588 | 0.709433337 | 0.710539441 | 0.684172216 | 0.70508933 |
| ResNet50 | 0.742686271 | 0.758786197 | 0.744189332 | 0.710686588 |  | 0.74705286 | 0.743972582 | 0.70292887 | 0.735757529 |
| VGG16 | 0.764111097 | 0.780587595 | 0.796563813 | 0.709433337 | 0.74705286 |  | 0.826759506 | 0.684326475 | 0.758404955 |
| VGG19 | 0.753960128 | 0.771494253 | 0.805089111 | 0.710539441 | 0.743972582 | 0.826759506 |  | 0.665797527 | 0.75394465 |
| Xception | 0.701109835 | 0.695546796 | 0.673512045 | 0.684172216 | 0.70292887 | 0.684326475 | 0.665797527 |  | 0.686770538 |

**Supplementary Table 3**. The average kappa coefficients of the all models and other models were over the acceptable threshold of 0.7.

| opponent | DenseNet121 | DenseNet169 | DenseNet201 | InceptionV3 | ResNet50 | VGG16 | VGG19 | average |
| --- | --- | --- | --- | --- | --- | --- | --- | --- |
| DenseNet121 |  | 0.780858159 | 0.791819015 | 0.708545801 | 0.742686271 | 0.764111097 | 0.753960128 | 0.756996745 |
| DenseNet169 | 0.780858159 |  | 0.798056885 | 0.703626402 | 0.758786197 | 0.780587595 | 0.771494253 | 0.765568248 |
| DenseNet201 | 0.791819015 | 0.798056885 |  | 0.708621525 | 0.744189332 | 0.796563813 | 0.805089111 | 0.774056614 |
| InceptionV3 | 0.708545801 | 0.703626402 | 0.708621525 |  | 0.710686588 | 0.709433337 | 0.710539441 | 0.708575516 |
| ResNet50 | 0.742686271 | 0.758786197 | 0.744189332 | 0.710686588 |  | 0.74705286 | 0.743972582 | 0.741228972 |
| VGG16 | 0.764111097 | 0.780587595 | 0.796563813 | 0.709433337 | 0.74705286 |  | 0.826759506 | 0.770751368 |
| VGG19 | 0.753960128 | 0.771494253 | 0.805089111 | 0.710539441 | 0.743972582 | 0.826759506 |  | 0.768635837 |

**Supplementary Table 4**. Multi-model combinations and kappa distance scores (KDS) for n = 2 to 7 models/combination.<0}

| N | Model | KDS (n, half) | KDS (n, least) | KDS (n, multi) |
| --- | --- | --- | --- | --- |
| 2 | ['DenseNet201', 'DenseNet121'] | 0.342 | 0.379 | −0.134 |
| 3 | ['DenseNet201', 'DenseNet121', 'VGG19'] | 0.060 | 0.533 | −1.513 |
| 4 | ['DenseNet201', 'DenseNet121', 'VGG19', 'DenseNet169'] | 0.256 | 0.636 | 0.938 |
| 5 | ['DenseNet201', 'DenseNet121', 'VGG19', 'DenseNet169', 'VGG16'] | 0.045 | 0.653 | −0.784 |
| 6 | ['DenseNet201', 'DenseNet121', 'VGG19', 'DenseNet169', 'VGG16', 'ResNet50'] | 0.185 | 0.759 | 1.183 |
| 7 | ['DenseNet201', 'DenseNet121', 'VGG19', 'DenseNet169', 'VGG16', 'ResNet50', 'InceptionV3'] | 0.049 | 0.803 | 0.308 |

KDS_(n,multi)_ becomes the highest when n = 6.<0}

**Supplementary Table 5**. {0>複合モデルの回答と血管占有面積比率<}0{>Responses of the multi-model system by grade and the area occupied by blood vessels<0}

|  | | Grade 0 | Grade 1 | Grade 2 | Grade3 | Sum |
| --- | --- | --- | --- | --- | --- | --- |
| Picked | N | 90 | 100 | 70 | 13 | 273 |
| Measurable* | N (％) | 62 (68.9) | 68 (68) | 58 (82.9) | 8 (61.5) | 196 (71.8) |
| Pixel coverage | ave (%) ± SD | 4.7 ± 2.8 | 9.1 ± 4.1 | 14.9 ± 5.8 | 25.6 ± 7.8 | - |

*The area occupied by blood vessels was measurable for about 72% of all images.<0}
